# Transcriptional Activation of OsDERF1 in *OsERF3* and *OsAP2-39* Negatively Modulates Ethylene Synthesis and Drought Tolerance in Rice

**DOI:** 10.1371/journal.pone.0025216

**Published:** 2011-09-26

**Authors:** Liyun Wan, Jianfei Zhang, Haiwen Zhang, Zhijin Zhang, Ruidang Quan, Shirong Zhou, Rongfeng Huang

**Affiliations:** 1 Biotechnology Research Institute, Chinese Academy of Agricultural Sciences, Beijing, China; 2 National Key Facility of Crop Gene Resources and Genetic Improvement, Beijing, China; 3 National Center for Plant Gene Research (Beijing), Beijing, China; University of Melbourne, Australia

## Abstract

The phytohormone ethylene is a key signaling molecule that regulates a variety of developmental processes and stress responses in plants. Transcriptional modulation is a pivotal process controlling ethylene synthesis, which further triggers the expression of stress-related genes and plant adaptation to stresses; however, it is unclear how this process is transcriptionally modulated in rice. In the present research, we report the transcriptional regulation of a novel rice ethylene response factor (ERF) in ethylene synthesis and drought tolerance. Through analysis of transcriptional data, one of the drought-responsive ERF genes, *OsDERF1*, was identified for its activation in response to drought, ethylene and abscisic acid. Transgenic plants overexpressing *OsDERF1* (OE) led to reduced tolerance to drought stress in rice at seedling stage, while knockdown of *OsDERF1* (RI) expression conferred enhanced tolerance at seedling and tillering stages. This regulation was supported by negative modulation in osmotic adjustment response. To elucidate the molecular basis of drought tolerance, we identified the target genes of OsDERF1 using the Affymetrix GeneChip, including the activation of cluster stress-related negative regulators such as ERF repressors. Biochemical and molecular approaches showed that OsDERF1 at least directly interacted with the GCC box in the promoters of ERF repressors *OsERF3* and *OsAP2-39*. Further investigations showed that OE seedlings had reduced expression (while RI lines showed enhanced expression) of ethylene synthesis genes, thereby resulting in changes in ethylene production. Moreover, overexpression of *OsERF3/OsAP2-39* suppressed ethylene synthesis. In addition, application of ACC recovered the drought-sensitive phenotype in the lines overexpressing *OsERF3*, showing that ethylene production contributed to drought response in rice. Thus our data reveal that a novel ERF transcriptional cascade modulates drought response through controlling the ethylene synthesis, deepening our understanding of the regulation of ERF proteins in ethylene related drought response.

## Introduction

Abiotic stresses, such as drought are primary factors that limit crop cultivation and yield worldwide. To cope with drought stress in rice (*Oryza sativa*), which consumes >70% of the water in agricultural areas – resulting in the acceleration of the shortage of the limited fresh water – recent research is applying integrated approaches including key genes that mediate plant responses to drought stress. For the characteristics that trigger global changes in drought-related genes, the application of transcription factor genes is becoming a powerful approach in the engineering of crop plants with enhanced tolerance to drought stress [1], [2]. Among the transcription factors, accumulating research shows that ethylene response factor (ERF) proteins function in plant growth, development and processes in response to biotic and abiotic stresses [2], [3], [4], [5], [6], [7], [8].

ERF proteins were identified as containing an ERF domain-conserved motif that binds to GCC box, DRE and other cis-acting elements [9], [10], [11], [12], acting as either a transcriptional activator or a repressor [13], [14]. For example, ERF activators CBF1/DREB2A, DREB1A and OsDREB1F enhance tolerance to salt, drought and low temperature in both rice and Arabidopsis [2], [15], whereas TSRF1 enhances pathogen resistance in tomato and tobacco [16]. Most importantly, ERF proteins affect stress responses through modulation of a given metabolism or synthesis pathway. For instance, Sub1A is an ERF-like protein that confers tolerance to submergence and drought in rice through affecting ethylene synthesis [4], [7]. The rice ERF proteins SNORKEL1 and SNORKEL2 can trigger remarkable internode elongation via gibberellins to avoid submergence [5]. Tomato JERF1 modulates the expression of an abscisic acid (ABA) biosynthesis-related gene that enhances tolerance to drought, salinity and cold in tobacco [17]. Moreover, investigations reveal that transcriptional repressors behave as negative regulators. For instance, ERF transcriptional repressors, AtERF4 and AtERF7 inhibit the expression of ethylene-responsive genes to decrease plant ethylene sensitivity through suppressing the expression of ABA-responsive genes [14], [18]. Importantly, the potato homolog of the tomato ERF transcriptional activator Pti4, which interacts with the repressor SEBF, represses the expression of potato *PR-10a* gene [19].

Increasing evidence reveals that the phytohormone ethylene is a key signaling molecule that regulates a variety of developmental processes and stress responses in plants [20], [21]. Enhancement of ethylene production initiates signaling with profound physiological consequences [22], [23]. Ethylene synthesis commences with methionine that is first converted to S-adenosylmethionine (S-AdoMet) by S-AdoMet synthetase, which is used to make the ethylene precursor 1-aminocyclopropane-1-carboxylate (ACC) by ACC synthase (ACS), a rate-limiting step in ethylene biosynthesis. Then ACC is oxidized by ACC oxidase (ACO) to generate ethylene in a reaction that also produces CO_2_ and hydrogen cyanide [24]. Ethylene biosynthesis is modulated by many factors or regulators at both transcriptional and post-transcriptional levels [25], [26], [27], [28]. For instance, ETO1 is an E3 ubiquitin ligase that targets type 2 ACS enzymes for degradation, thereby inhibiting its activity [22]. Analysis of protein turnover showed that ACS5 was more stable in *eto1* mutant seedlings than in wild-type seedlings [25], indicating that the mutation of E3 ligase in *eto1* does not degrade the ACS proteins. A member of the RING E3 ligase XBAT32 negatively modulates the abundance of ACS proteins and ethylene biosynthesis [29], [30], suggesting that modulation at the protein level is crucial for ethylene production. Moreover, transcriptional regulation plays a key role in ethylene biosynthesis. For example, during fruit ripening in tomato (*Solanum lycopersicum*), the expression of ACS and ACO genes stimulates the production of ethylene [26], [31], [32], [33], while cold, drought, salt and other abiotic stresses increase ACS transcripts and ethylene production [27]. In addition, transcription factors RIN, TERF1 and LeERF2 modulate the expression of ethylene biosynthesis genes and the downstream stress genes [12], [23], [34]. These investigations reveal that transcriptional modulation is a pivotal process in controlling ethylene synthesis, which further triggers the expression of stress-related genes and plant adaptation to stresses; however, it is unclear how this process is transcriptionally modulated in rice. In the present research, we report a novel ERF transcriptional activator that binds to GCC box to activate the expression of repressors, subsequently suppressing ethylene synthesis and reducing drought tolerance in rice. Therefore our data reveal a novel transcriptional cascade that involves the activation of multiple transcription factors that play antagonistic roles in gene regulation; and confirm from a different angle some recent discoveries on the positive role of ethylene in enhancing drought stress tolerance.

## Results

### OsDERF1 is a novel ERF protein of unknown function

Multiple factors, including water potential and osmotic adjustment, affect plant water status and drought tolerance. To elucidate the molecular basis underlying drought response, transcript levels of genes were screened under drought conditions. Using expression data for stress treatment in rice seedlings (http://www.ricearray.org), 12 drought-responsive ERF genes (DERF) were identified (Table S1, GEO accession number GSE26280). One of the DERF genes, *OsDERF1* (Os08g35240), located in rice chromosome 8, encodes a 243-amino-acid protein with predicted molecular mass of 23.8 kDa. Amino acid analysis indicated that the putative protein contains a typical ERF/AP2 domain at 26–91^th^ amino acids, an activation domain at 161–180^th^ amino acids, and a nuclear localization signal at 25–34^th^ amino acids. Comparison of the OsDERF1 amino acid sequences found little difference among 27 japonica and indica subspecies, except that in indica the 117^th^ Pro was instead Arg, and the 131^th^ Ser was missed (Figure S1). The conserved protein sequence implies that OsDERF1 might have an important regulatory role in rice.

### The expression of *OsDERF1* is induced by dehydration, ABA and ethylene precursor ACC

**Figure 1:**
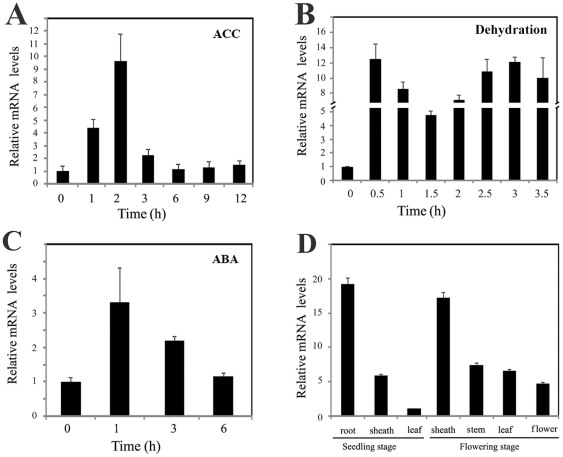
Expression of *OsDERF1* is responsive to dehydration, phytohormones and development stages. The expression of *OsDERF1* in Nipponbare in response to 500 µM ACC (A), dehydration (B), 100 µM ABA (C) and in different tissues (D). Transcripts of *OsDERF1* are indicated relative to the level of the control or a young leaf at seedling stage (taken as 1), referring to the transcripts of *OsActin1* in the same sample. Error bars (SD) are based on three independent experiments.

To reveal the regulatory roles of OsDERF1, we first analyzed the promoter sequence (2000 bp upstream of the transcription start site) by searching the promoter sequence against the PLACE database (http://www.dna.affrc.go.jp/PLACE/). Our analysis showed that *OsDERF1* contains multiple putative stress-responsive cis-acting elements including ABRE, MYC and MYB recognition sites. Then the transcripts of *OsDERF1* in response to dehydration, ABA and the ethylene precursor ACC were investigated with real-time PCR (Q-PCR) amplifications. Our results revealed that the expression of *OsDERF1* was highly induced (about fourfold) by ACC treatment at 1 h, and reached maximum induction at 2 h, then decreased (Figure 1A). And the transcripts of *OsDERF1* in response to dehydration were quickly peaked at 0.5 h then decreased. Interestingly, the transcripts of *OsDERF1* showed a second increase and peaked after dehydration induction for 3 h (Figure 1B). In addition, the expression of *OsDERF1* obviously increased and peaked after ABA treatment for 1 h (Figure 1C). Further analysis revealed that *OsDERF1* expression did not significantly alter when seedlings were subjected to salt and cold (data not shown), indicating that OsDERF1 might be associated with ethylene- and ABA-related drought response.

Next we further determined the expression of *OsDERF1* in different tissues of rice with Q-PCR. Our results showed that transcripts of *OsDERF1* were highly expressed in seedling roots and sheaths, compared to seedling leaves, whereas the expression of *OsDERF1* was mainly detected in leaves, sheaths, stems, and in flowers during flowering stages (Figure 1D), indicating that the OsDERF1 might mainly function at the tissues as the transcriptional expression.

Since OsDERF1 responded to dehydration, we tested the expression of *OsDERF1* in the upland rice Zhong Han3 and Brazil Triphibian, because these varieties are known to be drought tolerant [35], [36]. Surprisingly, we found that the expression level of *OsDERF1* was about 15% in Zhong Han3 and 10% in Brazil Triphibian, compared to that in Nipponbare under both normal growth conditions (Figure S2A). Even though drought stress significantly increased the expression of *OsDERF1* in Nipponbare, Zhong Han3 and Brazil Triphibian, the relative expression level of *OsDERF1* was only 20% and 30% in Zhong Han3 and Brazil Triphibian, respectively, compared to that in Nipponbare under drought growth conditions (Figure S2A), suggesting that the OsDERF1 might negatively regulate drought response.

### OsDERF1 is located in the nucleus and acts as a transcriptional activator

As discussed above, OsDERF1 is a novel putative ERF protein, and the characteristics of this transcription factor were analyzed. Firstly, the full length of *OsDERF1* was fused to GFP in pGDG vector in order to determine the subcellular localization of OsDERF1 in rice callus by *Agrobacterium tumefaciens* transformation. Our observations showed that GFP fluorescence in the callus cells transformed with pGDG vector (control) was distributed throughout the cells; however, after GFP was fused with OsDERF1, the fluorescence was seen exclusively in the nucleus. After fragment deletions, the data revealed that amino acids 25–34^th^ of OsDERF1 was a nuclear localization signal as predicted (Figure S3), demonstrating that OsDERF1 is a nuclear-localized protein.

**Figure 2:**
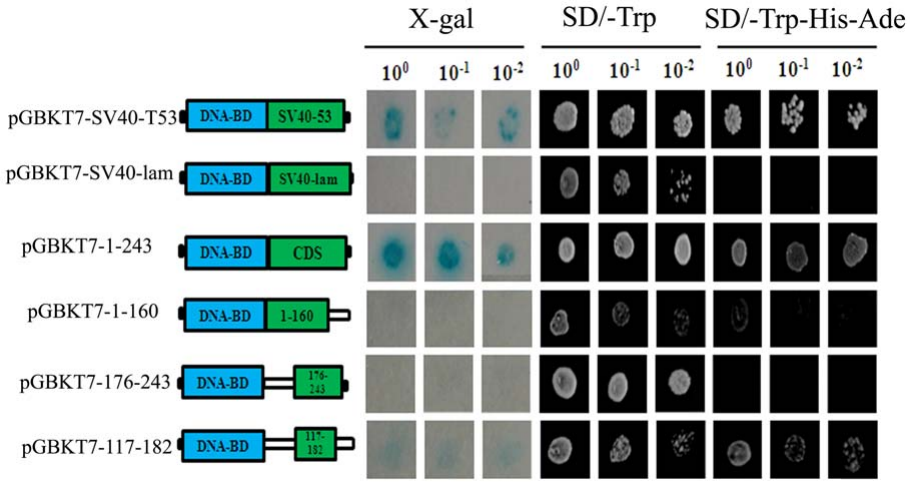
OsDERF1 contains a transcriptional activation domain. Left panel shows the schematic diagrams of various constructs used for transactivation described in Materials and Methods. DNA-BD indicates GAL4 DNA-binding domain; GAL4 AD for GAL4 activation domain; CDS and numbers for coding sequence length of OsDERF1. Right panel shows the β-galactosidase colony-lift filter assay and the yeast growth at different diluted concentration of the colony.

Next the full-length, the N-terminal of 160 amino acids, the C-terminal of 68 amino acids and the middle 66 amino acids of OsDERF1 were fused to the GAL4 DNA-binding domain, respectively, resulting in the plasmids of pGBKT7-1-243, pGBKT7-1-160, pGBK-176-243 and pGBK-117-182. The plasmids were then transformed into yeast strain AH109, with plasmids pGBK-GAL4-lam and pGBK-GAL4-SV40-T53 as negative and positive controls, respectively. The transformants of pGBK-GAL4-SV40-T53, pGBKT7-1-243 and pGBK-117-182 not only grew on the SD/-Trp medium, but also grew on the SD/-Trp –His-Ade medium and showed β-galactosidase activity, while the transformants of pGBKT7-1-160, pGBK-176-243 and the negative control pGBKGAL4-SV40-lam could not grow on the SD/-Trp –His-Ade medium and did not show β-galactosidase activity (Figure 2). These results indicated that OsDERF1 contains an activation domain in the amino acids 161–180, functioning as a transcriptional activator.

### OsDERF1 negatively regulates drought stress through affecting osmolyte accumulation in rice

**Figure 3:**
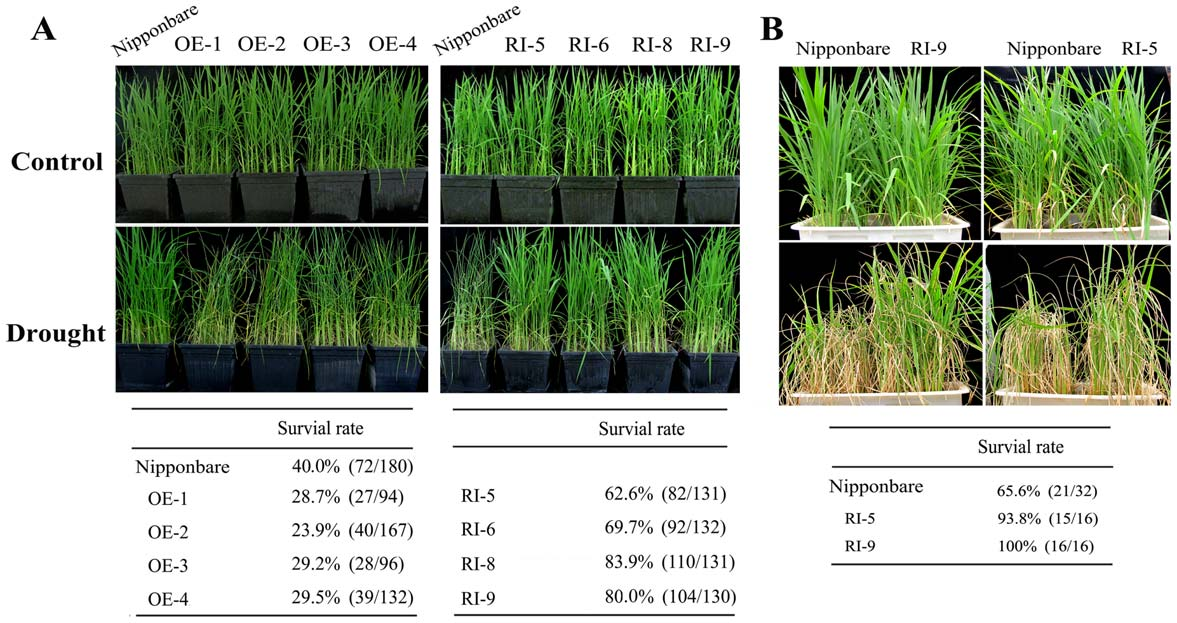
OsDERF1 is an important regulator for drought response. A. OE and RI plants were subjected to drought stress for 6 d and 8 d, respectively, during seedling stage. B. Tillering-stage plants were subjected to drought stress for 11 d and then allowed to recover for 9 d. The survival rate shows the percentage of recovered seedlings, compared to all plants used for the assay, after rewatering. And the numbers in parentheses shows the survival seedlings/total tested plants. Control: rice plants were grown under normal conditions. Drought: plants had the daily water supply withheld. Each experiment was repeated at least three times, with >30 seedlings each of Nipponbare and transgenic rice lines. Rice with reduced expression of *OsDERF1* denoted as RI, overexpressing *OsDERF1* rice as OE, and the different transgenic lines are indicated as the numbers.

To examine the regulatory function of OsDERF1, we generated overexpression of *OsDERF1* (OE) and RNA interference *OsDERF1* rice (RI), respectively. After Q-PCR analyses, the expression levels of *OsDERF1* that either decreased by less than half in the RI lines or obviously increased in OE lines, compared to Nipponbare (Figure S4A), were selected for our research. These transgenic lines did not show any obvious differences in plant development compared to Nipponbare at the seedling and tillering stages (Figure 3 and Figure S4B). After drought treatment for 6 d, the OE seedling leaves withered, while most Nipponbare plants still grew well. In contrast to OE lines, after drought treatment for 8 d, the control seedlings withered, whereas most RI lines grew well (Figure 3A). After all of the tested seedlings withered, the drought-treated seedlings were then allowed to recover by watering, giving survival rates of about 40% in Nipponbare, 60–84% in RI, and 24–30% in OE seedlings. Very interestingly, unlike the seedling stage, there were no differences between RI lines and Nipponbare plants after drought treatment for 11 d; however, after recovery for 9 d, the RI rice grew much better than Nipponbare (Figure 3B). These results indicated that OsDERF1 negatively regulated rice tolerance to drought, at least in the seedling and tillering stages.

Due to reputed drought tolerance [35], [36], we further compared the drought tolerance of RI-5 with the upland rice Zhong Han 3. Our observations showed that the RI rice performed similarly to Zhong Han 3 under drought stress, while Nipponbare was more sensitive to drought stress (Figure S2B and S2C), which displayed decreased expression of *OsDERF1* (Figure S2A). These results reflect that OsDERF1 might be an important regulator for drought response.

**Figure 4:**
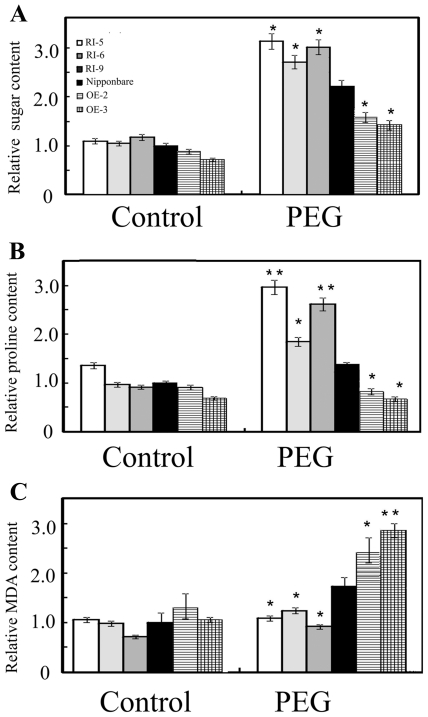
OsDERF1 modulates the generation of osmotic substances and MDA. A. Soluble sugar contents. B. Free proline. C. MDA content. Leaf tissues for measurements were sampled directly from two-week-old seedlings growing at 28°C (control) or after treatment with 15% PEG 6000 for 5 d. Data are the average of three replicates, and there were 10 plants per replicate. Error bars represent standard error (SE). Student's *t*-tests were conducted using the data in transgenic seedlings compared to these in Nipponbare at the same conditions. * and ** indicate significant difference at *P*<0.05 and *P*<0.01 probability, respectively.

It was reported that soluble sugars and proline are crucial factors in osmotic adjustment in plants [37], [38]. To determine whether OsDERF1 negatively modulates tolerance to drought through affecting osmolyte accumulation, we first measured the contents of soluble sugars and proline at seedling stage when grown under normal and PEG treatment. Our results showed that the there were not obvious changes among RI and OE lines under normal growth conditions. After PEG stress, although all the detected genotypes showed increase of the soluble sugars and proline contents, however, the relative increase rate was higher in RI seedling but lower in OE lines than these in Nipponbare (Figure 4A and 4B), suggesting that OsDERF1 reduces the accumulation of osmolytes to modulate drought tolerance. To reveal the mechanisms for decreased proline production, we analyzed the expression of a key enzyme gene involved in proline synthesis [39], [40], *OsP5CS* (*Os01g0848200*), by Q-PCR amplifications. As expected, the expression levels of *OsP5CS* were not obviously different between Nipponbare and RI lines under normal growth conditions. After PEG treatment, the transcripts of *OsP5CS* gene were up-regulated about 1.5- and 2.5-fold in the Nipponbare and RI lines, respectively, compared to normal conditions (Figure S5), indicating that OsDERF1 negatively regulated the expression of proline synthesis genes, resulting in the changes of proline, which may contribute to regulation of the drought response.

Increasing research indicates that abiotic stresses result in free-radical production and cellular membrane injury [41], [42], [43], [44]. Our investigations also showed that RI seedling significantly decreased while OE lines enhanced malondialdehyde (MDA) accumulation under PEG treatment (Figure 4C), an end product of membrane lipid peroxidation, indicating that OsDERF1 positive affects the production of oxidative stress.

### OsDERF1 dramatically alters the expression profiles

**Figure 5:**
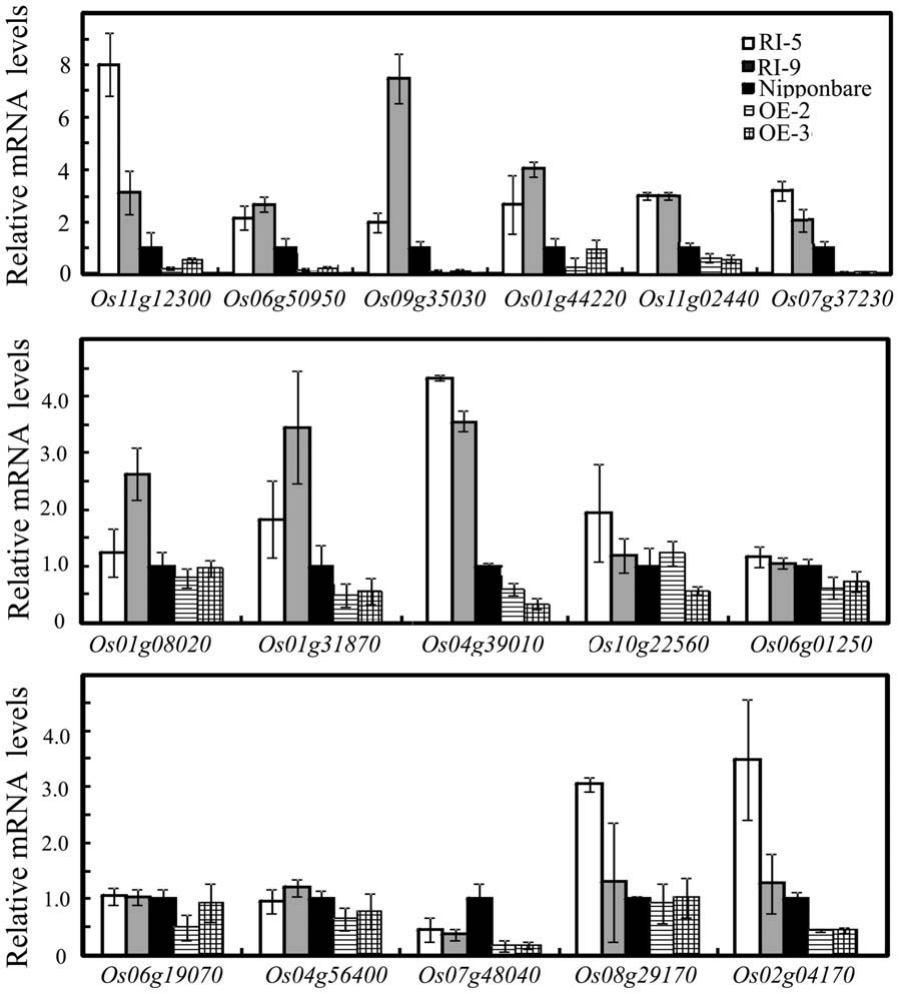
OsDERF1 modulates the expression of stress-related genes in rice. Selective confirmation of the OsDERF1 upstream-regulated candidate genes in Affymetrix GeneChip using Q-PCR amplifications. The putative functions related to stress response of 16 representative genes are discussed in the text. Transcripts of each gene are indicated relative to the level of the Nipponbare (taken as 1), referring to the transcripts of *OsActin1* in the same sample. Error bars (SD) are based on three independent experiments.

To elucidate the molecular basis of drought tolerance caused by OsDERF1, we identified the target genes of OsDERF1 through the genome-wide expression profile changes in the Nipponbare and OE lines using the Affymetrix GeneChip under normal growth conditions at the four-leaf stage. Our analysis revealed that there are about 1491 genes in overexpressing *OsDEF1* rice that showed two-fold changes, compared to these in Nipponbare. 512 of the genes were lowered the expression in the OE lines (Table S2a), which near half of the putative-regulated genes were annotated or shown to be involved in stress response (Table S2b). Examples were genes encoding cytochrome family proteins, chlorophyll synthesis-related, transporter, secondary metabolites synthesis related and stress-related regulatory factors. 16 of the down-regulated stress-related genes were confirmed with Q-PCR. These putative stress-related genes include NBS-LRR disease resistance protein (*Os11g12300*), lipolytic enzyme (*Os06g50950*), inositol monophosphatase (*Os07g37230*), chalcone–flavanone isomerase *(Os11g02440)*, ADP-glucose pyrophosphorylase (*Os01g44220*), DRE-binding protein 1A (*Os09g35030*), transmembrane transporter protein (*Os01g08020*, *Os01g31870*, *Os04g39010* and *Os10g22560*), cytochrome P450 family protein (*Os06g01250* and *Os06g19070*), Glutamine synthetase (*Os04g56400*), peroxidase (*Os07g48040*), quinone oxidoreductase-like protein (*Os08g29170*) and L-aspartate oxidase (*Os02g04170*). Our results showed that the expression levels of 9 of the selected 16 genes (*Os11g12300*, *Os06g50950*, *Os09g35030*, *Os01g44220*, *Os11g02440*, *Os07g37230*, *Os01g31870*, *Os04g39010* and *Os02g04170*) were significantly increased in the RI lines but decreased in OE lines based on Q-PCR analyses, while the expression of the other genes did not have consistent changes in OE and RI lines (Figure 5). We also searched the ethylene synthesis-related genes in the microarray data, and found that ACO and ACS genes were changed in the OE seedlings (Table S2c) – implying that OsDERF1 might affect ethylene biosynthesis in rice.

**Figure 6:**
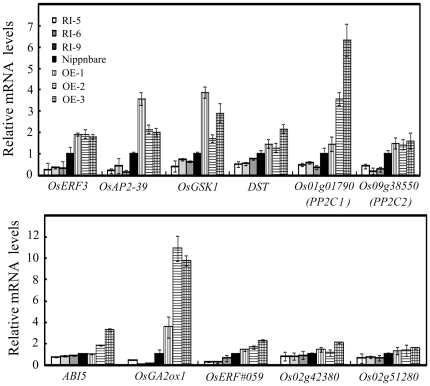
OsDERF1 transcriptionally activates the expression of putative repressors in rice. Confirmation of the OsDERF1 putative target genes obtained from the data of Affymetrix GeneChip using Q-PCR amplifications. The putative functions of the 11 putative repressors are discussed in the text. Transcripts of each gene are indicated relative to the level of the Nipponbare (taken as 1), referring to the transcripts of *OsActin1* in the same sample. Error bars (SD) are based on three independent experiments.

Interestingly, 979 genes (1070 probes) showed two fold increases in OE lines compared to Nipponbare (Table S3a), suggesting that overexpression of *OsDERF1* can activate the expression of a large number of genes. Although the predicted functions of the up-regulated genes are extremely diverse (Table S3b), there were many stress-related genes (e.g. glutathione s-transferases) and especially stress-related negative regulator genes (Table S3c), including brassinosteroid signaling OsGSK1 [45], DST zinc finger transcription factor [46], ABA signaling regulators ABI5 [47] and PP2Cs [48], [49], [50], gibberellin synthesis and accumulation regulator OsGA2ox1 [51], [52], shoot meristem differentiation regulators OsTCPs [53] and ERF repressors (*OsERF#075/OsERF3/Os01g58420*, *OsERF#077/OsAP2-39/Os04g52090* and *OsERF#059/Os10g25170*) [8], [9], [54], [55]. In order to investigate whether OsDERF1 can activate the expression of the repressor genes, the expression levels of the selected 11 genes were analyzed in the OE and RI lines. Our results showed that 8 of the genes (*OsERF3*, *OsAP2-39*, *DST*, *GSK*, *PP2C1/2*, *OsABI5* and *OsGA2ox1*), except *OsERF#59*, *Os02g42380* and *Os02g51280*, were activated in the OE lines, but suppressed in RI lines (Figure 6), suggested that OsDERF1 was required at least for the expression of the negative regulators.

### OsDERF1 directly binds to the promoters of ERF repressor genes

**Figure 7:**
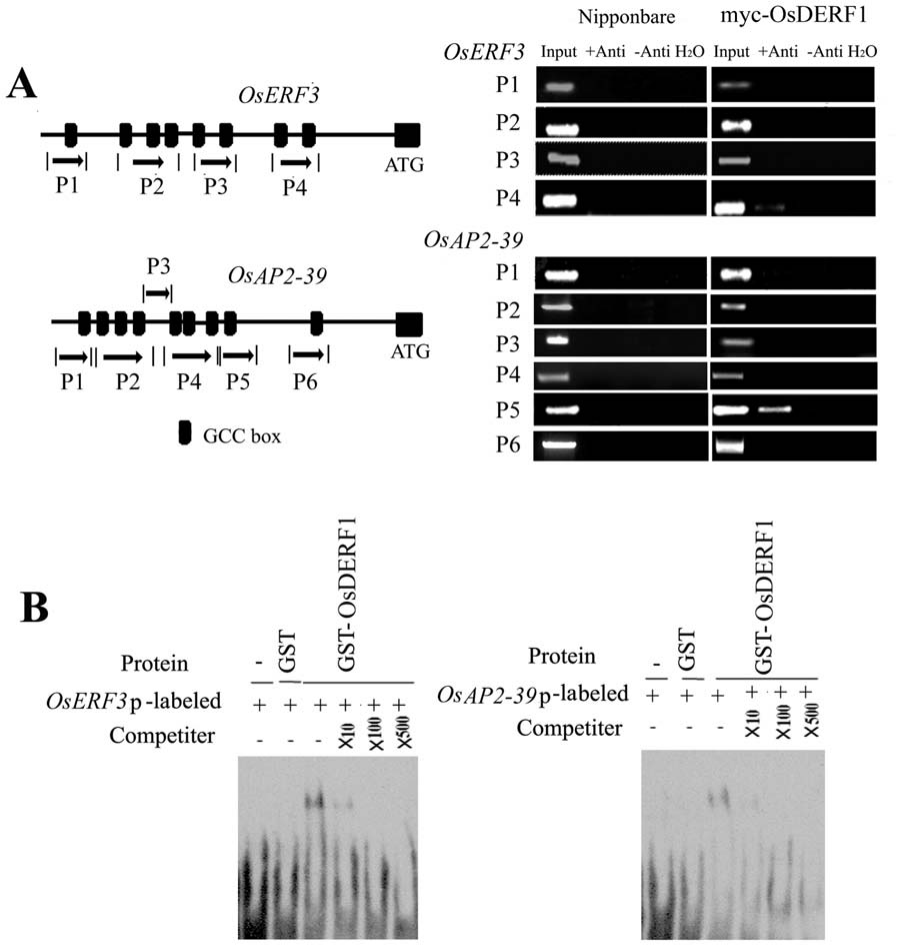
OsDERF1 interacts with the promoters of ERF repressors *in vivo* and *in vitro*. (A) The interaction of OsDERF1 with the promoters of ERF repressors in plants. Left panel shows the fragments of promoters of ERF repressors *OsERF3* and *OsAP2-39*, which were used for PCR amplifications. Numbers indicate the positions of the nucleotides at the 5′- or 3′-ends of each fragment relative to the translation start site. Right panel presents PCR amplification samples before input and after immunoprecipitation with (+) or without (−) antibody myc (Anti) detected using specific primers in Nipponbare and transgenic seedlings with 35S-myc-OsDERF1. (B) Detection of the interaction of OsDERF1 with GCC box in *OsERF3-*p4 and *OsAP2-39*-p5 promoters using EMSA. Probe sequences of GCC box from the two ERF repressor promoters are given in Table S4. The detection of probes after reaction with GST protein was taken as a negative control. Competition assays were processed using unlabeled probes after reaction with GST–OsDERF1 in the presence of labeled probe GCC box.

Because of the transcriptional activation of OsDERF1 in the expression of ERF repressor genes (Figure 6), we further elucidated whether OsDERF1 directly activated the expression of these ERF repressor genes. After analyzing the promoter sequences of *OsERF3* and *OsAP2-39* using PLACE, we found that the promoters of *OsERF3* and *OsAP2-39* contain 8–9 GCC boxes (Figure 7A). Using chromatin immunoprecipitation (ChIP) assays, we detected that OsDERF1 bound to the promoters of *OsERF3/OsAP2-39 in vivo*. In this system, we used 35S-myc-OsDERF1 transgenic rice and Nipponbare plants. Specific immunoprecipitation was conducted with an anti-myc antibody. The promoter fragments *OsERF3*-p4 and *OsAP2-39*-p5, which contain GCC box, were amplified from the anti-myc immunoprecipitates of 35S-myc-OsDERF1 extracts (Figure 7A). In the Nipponbare seedlings that there was no myc-tagged protein expressed, no DNA fragment could be detected from anti-myc immunoprecipitates, evidence of the specific binding of OsDERF1 to these promoters *in vivo*.

Next an electrophoretic mobility shift assay (EMSA) was carried out to test whether OsDERF1 physically bound to the cis-acting elements of the GCC box *in vitro*. In this assay, purified GST protein was isolated as a control. The sequences of GCC box from *OsERF3*-p4 and *OsAP2-39*-p5, respectively, were used for probes (Table S4). Our results showed that OsDERF1 protein caused a mobility shift in the labeled probes from the GCC box of *OsERF3* and *OsAP2-39*, which migrated more slowly than the free probes, whereas the GST control did not (Figure 7B). To further investigate the specific binding, competition assay was performed. Our EMSA showed that the binding activity of OsDERF1 was completely inhibited with excess of unlabeled *OsERF3*-p4 or *OsAP2-39*-p5 (Figure 7B), indicating that OsDERF1 directly interacted with GCC box in *OsERF3* and *OsAP2-39* genes to activate the genes' expression.

### Transcriptional activation of OsDERF1 in *OsERF3* and *OsAP2-39* negatively regulates ethylene biosynthesis

**Figure 8:**
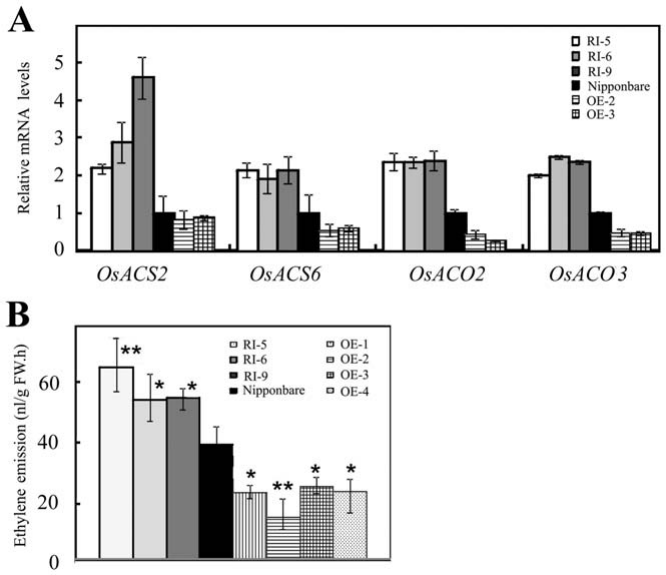
Alteration of the *OsDERF1* transcripts causes changes in production of ethylene through regulating the expression of ethylene biosynthesis genes. A. Expression of ACS and ACO genes detected by Q-PCR. The expression level of each gene in the Nipponbare was standardized to 1, referring to the internal control of *OsActin1*. B. Measurement of ethylene emission. The data, average of three independent biological assays plus SD, show ethylene production in terms of the increase over controls (sealed vials without any seedlings). nl/gFW.h indicates the amount of ethylene per gram fresh weight seedling in an hour. Student's *t*-tests were conducted using the data in transgenic seedlings compared to these in Nipponbare. * and ** indicate significant difference at *P*<0.05 and *P*<0.01 probability, respectively.

**Figure 9:**
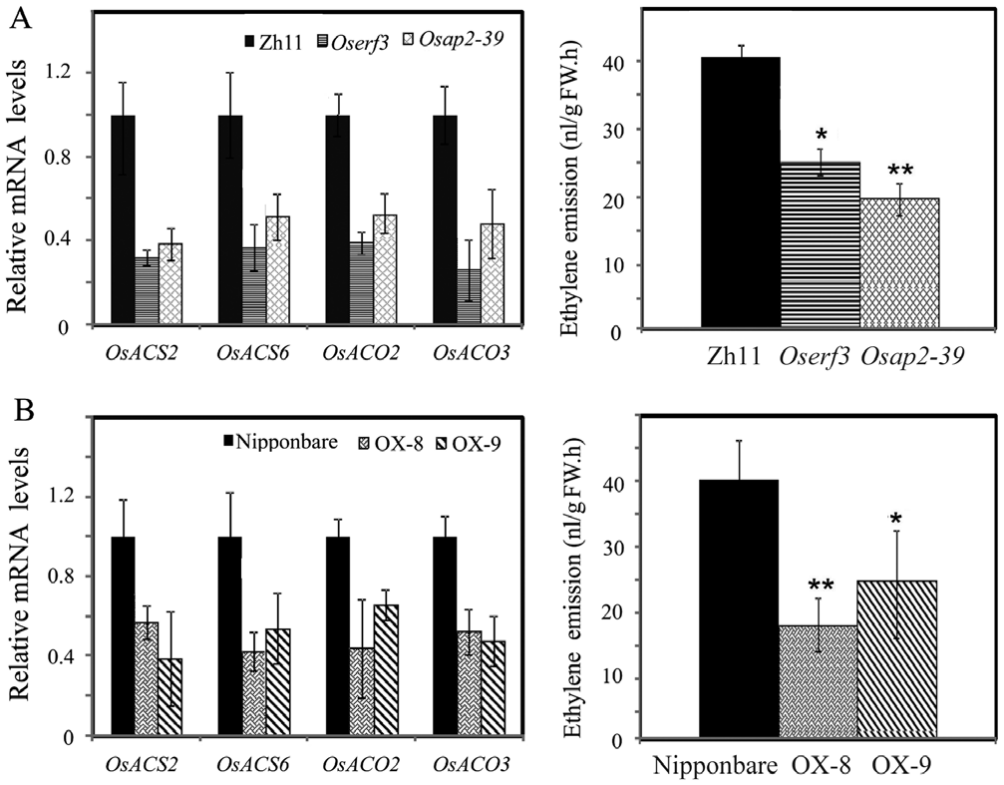
Overexpressor mutants *Oserf3* and *Osap2-39* display decreased expression of ethylene biosynthesis genes and ethylene production. Detection of the expression of ethylene biosynthesis genes detected by Q-PCR and ethylene emission in overexpressor mutants (A) and in overexpressing *OsERF3* transgenic lines (B). The expression level of each gene in the Nipponbare or Zhonghua 11 (Zh11) was standardized to 1, referring to the internal control of *OsActin1*. *Oserf3* and *Osap2-39* are activation-tagged T-DNA insertion mutants in Zh11. The ethylene emission, average of three independent biological assays plus SD, is in terms of the increase over controls (sealed vials without any seedlings). nl/gFW.h indicates the amount of ethylene per gram fresh weight seedling in an hour. Student's *t*-tests were conducted using the data in transgenic seedlings compared to these in Nipponbare. * and ** indicate significant difference at *P*<0.05 and *P*<0.01 probability, respectively.

To examine the regulatory relationship of *OsDERF1*, *OsERF3* and *OsAP2-39*, we first identified overexpressor lines of *OsERF3* and overexpressing mutants *Oserf3* and *Osap2-39* (Figure S6), which are activation-tagged T-DNA insertion mutants in Zhonghua 11. Since OsDERF1 is an ethylene-responsive transcriptional activator that activates the expression of ERF repressors, the expression of rice ethylene biosynthesis genes was firstly analyzed, which contain GCC box or DRE in their promoters (Table S5). Results showed that four of the ACS genes (*OsACS2* and *OsACS6*) and ACO genes (*OsACO2* and *OsACO3*) were suppressed in OE-2 and OE-3 lines, consistent with our analysis of microarray (Table S2c), but enhanced in RI-5, -6 and -9 lines, compared to these in Nipponbare (Figure 8A). For the activation of OsDERF1 in *OsERF3* and *OsAP2-39*, we then further checked the expression of OsDERF1-regulated ACS and ACO genes in *Oserf3*- and *Osap2-39*-overexpressor mutants. Our research indicated that overexpression of these repressor genes decreased the expression of the ACS and ACO genes (Figure 9A), suggesting that OsDERF1 at least partly targets ERF repressors to suppress ethylene synthesis genes.

To confirm whether the suppression of the OsDERF1–ERF complex in ethylene synthesis genes affects ethylene production, we measured the ethylene contents using gas chromatography. Our data showed that the production of ethylene decreased about 50% in OE rice, but increased about 60% in RI lines, compared to Nipponbare seedlings (Figure 8B). Student's *t*-tests indicated significant differences in production of ethylene between Nipponbare and OE and RI seedlings. Most importantly, *Oserf3-* and *Osap2-39*-overexpressor mutants displayed reduced ethylene production (Figure 9A). Therefore, our results probably indicate that OsDERF1 negatively regulated ethylene production through transcriptional activation of ERF repressors.

### ACC application partially recovers the drought tolerance of OsDERF1-targeted *OsERF3*

**Figure 10:**
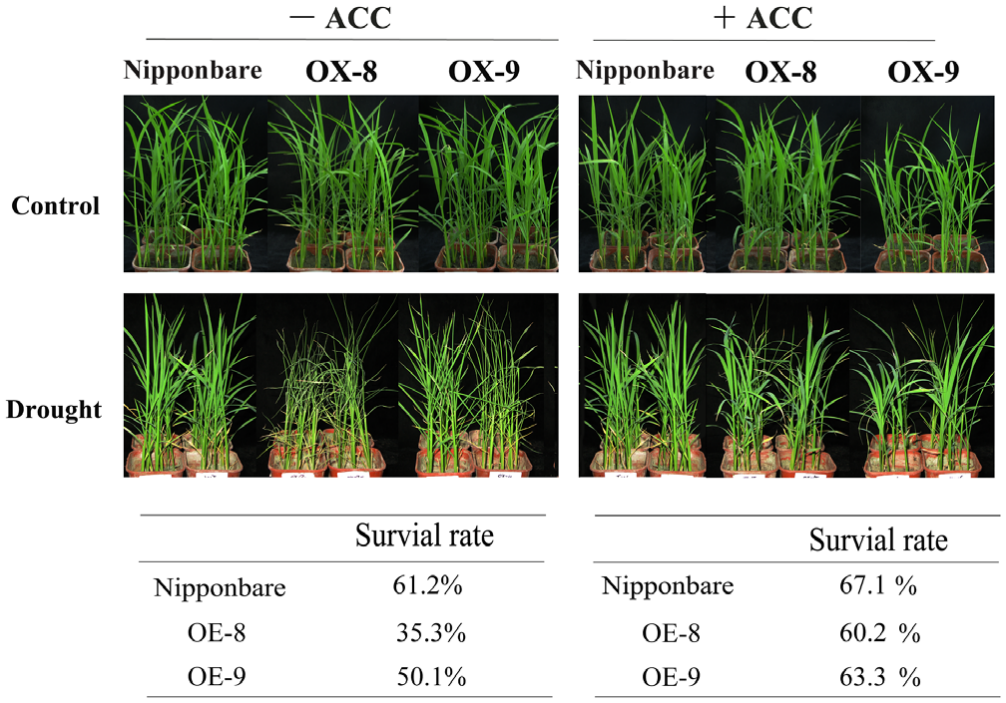
Overexpression of *OsERF3* causes drought sensitivity and ACC application reversed this response. A. Drought stress treatment during seedling stage. Plants were subjected to drought stress for 7 d with or without 200 mM ACC application. The percentage of survival of seedlings was calculated after drought-treated plants were allowed to recover for 10 d. Control: rice plants were grown under normal conditions. Drought: plants had daily water supply withheld. +/−ACC: plants were pretreated with 200 µM ACC or same amount water then were withheld daily water supply. Each experiment was repeated at least three times, with >36 seedlings each of Nipponbare and transgenic lines (OX-8 and -9).

As discussed above, OsDERF1 activates the expression of ERF repressors and negatively modulates drought tolerance, and we questioned whether ERF repressors *OsERF3* and *OsAP2-39* regulate drought response. To address this, we first checked the response of *OsERF3*- and *OsAP2-39*-overexpressor mutants under drought response. Our observations revealed that overexpression of the repressor gene reduced drought tolerance, compared to Nipponbare (Figure S7). Next we found that overexpression of *OsERF3* in Nipponbare (OX) significantly decreased the expression of ethylene synthesis genes and ethylene emission (Figure 9B), similar to the regulation of *OsERF3*- overexpressor mutant. Then we further compared whether the drought response is associated with ethylene reduction. In this case, we used Nipponbare and overexpressing *OsERF3* lines. Our observation confirmed that OX lines were more sensitive to drought than Nipponbare did. Most importantly, application of ACC greatly recovered the drought-sensitive phenotype (Figure 10). After all Nipponbare seedlings had withered, we rewatered the drought-treated seedlings and determined the survival rates: 61.2% in Nipponbare and 35–50% in OX lines without ACC pretreatment. After ACC application the survival rate increased to 67.1% in Nipponbare and 60–63% in OX lines, suggesting that ethylene application improved drought tolerance. Thus our data indicate that the ethylene synthesis contributed to the drought tolerant phenotype.

## Discussion

Ethylene is an important regulator of plant development and stress response, and transcriptional modulation has a pivotal role in the regulation of ethylene synthesis that modulates the expression of a series of stress-related genes and plant adaptation to stresses. In the present research, we reported that a novel ERF transcriptional activator at least modulated the expression of ERF repressors *OsERF3* and *OsAP2-39* in rice, evidencing a novel transcriptional cascade that involves the activation of multiple transcription factors that play antagonistic roles in gene regulation. Interestingly, overexpression of the ERF repressors suppressed ethylene production, revealing a novel fact that ERF genes induced by ABA and dehydration suppress ethylene production and enhance drought stress tolerance. Furthermore, the application of ACC recovered the drought-sensitive phenotype in transgenic lines overexpressing *OsERF3*, demonstrating that the reduced ethylene production caused by the OsDERF1–ERF complex might contribute to the drought response in rice. Thus our data in the present research confirmed from different angle some recent discoveries on the positive role of ethylene in enhancing drought stress tolerance, deepening our understanding of regulation of ERF protein in ethylene related drought response.

ERF proteins play important roles in plant response to biotic and abiotic stress and in development and metabolism in rice [5], [7], [8], [15]. For example, ERF protein OsAP2-39 controls the ABA/gibberellins balance in rice, which in turn regulates plant growth and seed production [8]. Moreover, tomato ERF transcriptional activator LeERF2 activates ethylene biosynthesis genes to control ethylene production in tomato and tobacco [12], while ERF repressor SlAP2a is a negative regulator of fruit ripening through repressing the expression of *ACS2*, *ACS4* and *ACO1* [3]. This transcriptional regulation is based on the ability of ERF proteins to bind to different DNA fragments that regulate the expression of different downstream genes [12], [56], [57], [58], [59]. The present study revealed that the ERF protein OsDERF1 interacted with GCC box present in the promoters of transcriptional repressors including *OsERF3* and *OsAP2-39*, which consequently suppressed the expression of ethylene synthesis possibly through transcriptional binding to GCC box and DRE (Figure S8), consistent with the regulation of other ERF repressors [9]. Therefore, our data reveal that an ERF transcriptional cascade negatively modulates drought response through controlling ethylene biosynthesis. In addition, this regulation was attributed to negative modulation in osmotic adjustment, which affects the status of lipid peroxidation under drought stress. More interestingly, both *SUB1A* and *OsDERF1* are inducible by drought and ethylene and negatively regulate ethylene production, but the difference is that SUB1A is a positive while OsDERF1 is a negative regulator in drought response, distinguishing the regulation of Sub1A and OsDERF1 in drought tolerance in rice [4], [7]. Noticeably, the ethylene content in drought tolerance Zhong Han 3 and Brazil Triphibian [35], [36] was differential, although both displayed decreased expression of *OsDERF1*. Our analysis revealed that Zhong Han 3 had a higher ethylene emission than Nipponbare under normal growth conditions (Figure S2B), increasing our understanding of ERF regulation in ethylene related drought response. But Brazil Triphibian had no obvious different ethylene production with Nipponbare (Figure S2B), implying that multiple pathways, rather than ethylene signaling, are involved in the regulation of drought response.

OsDERF1 can not only activate the expression of rice ERF repressors, but can also up-regulate the expression of *OsGSK1*, *OsPP2C*, *OsABI5* and *OsGA2ox1*. It was reported that *OsGSK1* (*Oryza sativa* glycogen synthase kinase3-likegene 1) is a member of the plant GSK3/SHAGGY-like protein kinase genes and an ortholog of the Arabidopsis brassinosteroid insensitive 2 (*BIN2*). Knockout of *OsGSK1* showed enhanced tolerance to cold, heat, salt and drought stresses, whereas overexpression of *OsGSK1* led to a stunted growth phenotype, suggesting that OsGSK1 might serve as a negative regulator of brassinosteroid-signaling [45]. The overexpression of *OsABI5* could rescue ABA-insensitivity of *abi5*-1during seed germination, showing ABA hypersensitivity, while repression of *OsABI5* promoted stress tolerance and resulted in low fertility of rice [47]. It is well known that PP2Cs act as negative regulators of ABA signaling [48], [49], [50], whereas OsGA2ox1 is a negative regulator in gibberellin synthesis and accumulation of gibberellins1 [51], [52]. Moreover, OsAP2-39 controls the ABA/gibberellin balance in rice by regulation of both ABA biosynthesis and gibberellin metabolism in rice [8]. The present study showed that OsAP2-39 can affect ethylene synthesis by regulating the expression of ACS and ACO genes. For direct transcriptional modulation of OsDERF1 on *OsAP2-39*, it is possible that OsDERF1 has multiple regulatory effects in plant development and drought response by controlling the balance of ABA, gibberellins and ethylene, transcriptionally integrating multiple hormone homeostasis in rice.

Increasing amounts of research have shown that improved stress tolerance can be accompanied by reduced yield, biomass or plant growth under normal conditions [60], [61], [62]. For instance, rice seedlings overexpressing *OsNAC6* were smaller than controls and their reproductive yields were likewise lower than controls [61]. *OsMYB3R-2* overexpressing plants had shorter roots and retarded growth [60]; while the overexpression of Arabidopsis *TINY* showed shorter plant height, shorter hypocotyl elongation, and reduced fertility [62]. In the present research, neither overexpression of *OsDERF1* nor RI lines affect rice growth and yield, compared to Nipponbare (Data not shown), providing a cue for genetic/biotechnological modifications of stress-tolerant crops.

**Figure 11:**
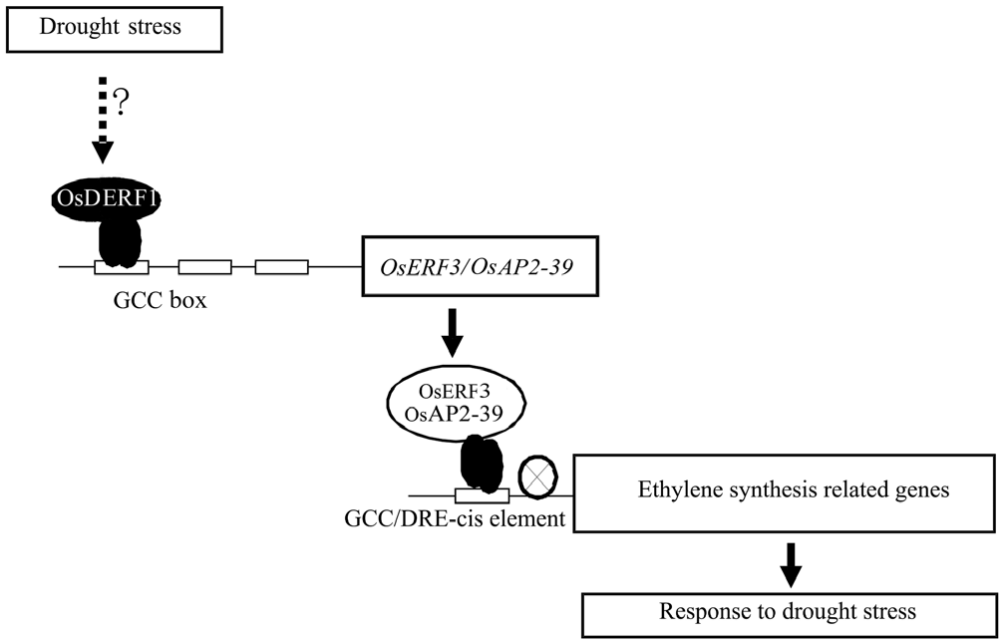
OsDERF1 transcriptionally regulates drought response in rice. Transcriptional activator OsDERF1 directly activates the expression of repressor genes, such as *OsERF3* and *OsAP2-39*. The repressors in turn further repress the expression of ethylene synthesis-related genes, possibly interacting with GCC box and DRE, resulting in the reduction of ethylene production and drought sensitivity in rice.

Based on the above discussion, we suggest that ERF protein OsDERF1 as a transcriptional activator directly interacts with GCC box, resulting in the expression of rice repressors *OsERF3* and *OsAP2-39* (Figure 11). These repressors further suppress the expression of ethylene synthesis-related genes, possibly interacting with GCC box and DRE, thereby reducing ethylene production. The decreased ethylene production disorders the hormone balance, subsequently affecting osmotic adjustment and drought response in rice. Thus, the present study demonstrates for the first time that an ERF transcriptional complex modulates drought response through controlling ethylene synthesis.

## Materials and Methods

### Plant materials and growth conditions

Rice (*Oryza sativa*) seeds of Zhonghan 3 and Brazil Triphibian were gift from Dr. H. Lin (Institute of Plant Physiology and Ecology, Chinese Academy of Sciences). The overexpressor mutants of *Oserf3* (SHIP NE2275) and *Osap2-39* (SHIP NE2293) were obtained from Shanghai T-DNA Insertion Population (http://ship.plantsignal.cn), which are activation-tagged T-DNA insertion mutants in Zhonghua 11. Other rice cultivars and transgenic rice were from our laboratory. After seed germination, seedlings were transplanted in sandy soil in growth chambers at 25°C with a 14/10 h light/dark cycle. 4-week-old rice seedlings or flowing mature plants were used for gene expression analyses as described by [63]. For ABA and ACC treatments, rice plants were sprayed with 100 µM ABA and 500 µM ACC, respectively, until the solution to drop off from the seedlings, for the indicated time. ABA was dissolved in ethanol, and the same concentration of 0.01% (v/v) ethanol only was used for controls. For dehydration treatment, the rice plants were removed from soil, and washed with water. Then the seedlings were treated in the air without water supply for the indicated time interval.

### RNA isolation and real-time PCR amplifications

Total RNAs were isolated from rice leaves using TRIzol reagent (Tiangen, China). For Q-PCR analysis, first-strand cDNAs were synthesized from DNaseI-treated total RNAs using MLV reverse transcriptase (Invitrogen, USA) according to the manufacturer's instructions. Q-PCR amplifications were performed on a 96-well plate with a Biorad IQ5 real-time PCR system. Each reaction contained 10 mL of SYBR Green Master Mix Reagent (Takara, Japan), 1.0 µL of cDNA samples and 200 nM gene-specific primers (Table S4) in a final volume of 20 µL. The rice *OsActin1* gene was used as an internal control. The relative expression levels were determined as described [64].

### Transactivation assay of OsDERF1

The different lengths of *OsDERF1* (including activating domain) were fused in-frame to the DNA-binding domain vector pGBKT7, respectively. The fusion plasmids were transformed into AH109 with lacZ as described by the manufacturer (Clontech, USA). The transformants were selected by growth on selective medium plates at 30°C for 3 d. The colony lift filter assay, using o-nitrophenyl β-D-galactopyranoside as a substrate, was performed subsequently to determine the ability of each translation product to activate transcription.

### Subcellular localization of OsDERF1

For subcellular localization, the different lengths of *OsDERF1* coding area without the termination codon were amplified by PCR with specific primers (Table S4). The fragments were introduced into the pGDG vector, resulting in an in-frame fusion between the GFP gene and different lengths of *OsDERF1* cDNA. The different fusion constructs and control (pGDG) were separately transformed into rice callus by *Agrobacterium tumefaciens*-mediated transformation. After incubation of transformed rice callus for 48–72 h at 28°C, the localization of the fusion protein was detected by confocal fluorescence microscopy (Leica SP2, Germany).

### Generation of transgenic plants

The full-length cDNAs of *OsDERF1* and *OsERF3* were separately amplified by PCR using specific primers (Table S4) and cloned into pCAMBIA1307 (a derivative of pCAMBIA1300 carrying the 2*CaMV 35S promoter and the OCS terminator). To determine the essential roles of OsDERF1 in rice, we used an RNA interference approach to knockdown *OsDERF1*, driven by the rice *Actin1* promoter as described [65]. Because OsDERF1 is a member of a large ERF subfamily [55], we used the less conserved region at the C-terminus (localized at amino acids 173–243), to interfere with the expression of *OsDERF1* (Table S4). Then the resulting plasmids were separately introduced into rice (cv. Nipponbare) using *Agrobacterium*-mediated transformation. Transformed plants were selected on the basis of their resistance to hygromycin or G418. The efficiency and specific interference in RNA interference lines were confirmed using Q-PCR. In the present study, only the transgenic lines that the reduced expression of *OsDERF1* was >50% and that did not affect the expression of homologue genes were used (Figure S4A). Rice with reduced expression of *OsDERF1* was denoted as RI, overexpressing *OsDERF1 and OsERF3* rice as OE and OX, respectively, and the different transgenic lines are indicated as the numbers.

### Drought treatment

Seeds of different genotypes were firstly germinated at 30°C, and then transferred to pots containing soil and grown in greenhouse at 28°C and 16/8 h light/dark. For the drought treatment, rice plants were exposed to progressive drought by withholding water until the drought phenotype was observed. For ACC treatment, plants were first pretreated with 200 µM ACC or same amount water then were withheld daily water supply.

### Measurement of soluble sugar, proline and malondialdehyde

After being subjected to PEG treatment for 5 d, the plants were analyzed for proline, soluble sugar contents and malondialdehyde (MDA). For the determination of proline, 0.5 g of seedling tissue was homogenized in 2 mL of 3% aqueous sulfosalicylic acid and centrifuged. Proline content was determined as described [42]. Total soluble sugar concentrations were estimated according to [66], using about 20 mg leaf tissues. And soluble sugar was calculated using glucose curves as a standard. MDA equivalents were measured as described [43]. The results were reported as content relative to that of Nipponbare rice.

### Conduction and analyses of GeneCHIP

Hybridization with Affymetrix GeneChip Rice Genome Arrays was performed at CapitalBio Corporation. Three independent biological replicates of 4-week-old overexpressing *OsDERF1* transgenic and Nipponbare seedlings were used for microarray experiments. For microarray analysis, the experimental process was followed the standard protocol of Affymetrix GeneChip service (CapitalBio). To find differentially expressed genes, the change fold of each gene between overexpressing *OsDERF1* transgenic and Nipponbare plants was calculated by SAM (TM) software. Genes exhibiting more than 2-fold enhanced or 2-fold reduced at transcription level and Q-value less than 5% were listed in Table S2 and S3. CapitalBio MAS3 system was used for the molecular function enrichment and pathway analyses.

### Chromatin immunoprecipitation

Chromatin immunoprecipitation was conducted as described [67]. Briefly, rice seedlings (2 g) of overexpressing *myc-OsDERF1* fusion using pCAMBIA1307–myc–OsDERF1 and Nipponbare as control were fixed with 1% formaldehyde in phosphate-buffered saline (PBS) at room temperature for 10 min with gentle agitation. After two washes with 40 mL ice-cold PBS, the samples were ground to extract the proteins and DNA. The chromatin solution was then sonicated to shear the DNA into fragments. After centrifuging, the chromatin pellet was re-suspended in 300 µL buffer containing 50 mM Tris-HCl (pH 8.0), 10 mM EDTA, 1% SDS, 1 mM PMSF (phenylmethanesulfonyl fluoride) and protease inhibitors. The above solution was divided into three portions and then 900 µL buffer [1.1% Triton X-100, 1.2 mM EDTA, 16.7 mM Tris-HCl (pH 8.0) and 167 mM NaCl] was added. After adding 40 µL of salmon sperm-sheared DNA to each chromatin sample with gentle agitation and overnight incubation at 4°C, the pellet was rinsed three times and re-suspended in a buffer containing 50 mM Na_3_PO_4_ (pH 8.0), 167 mM NaCl, 10 mM imidazole and protease inhibitors. The input solution was then used for performing immunoprecipitation using anti-myc antibodies. The DNA fragments were cleaned up using a PCR DNA purification kit (Tiangen, China). Promoter fragments were amplified using 1 µL of purified DNA as a template in each PCR reaction. Primer sequences for ChIP PCR experiments are provided in Table S4.

### Preparation of fusion protein and gel mobility shift assay

To analyze the DNA-binding specificity of OsDERF1 *in vitro*, full length cDNA sequence of *OsDERF1* was first cloned into the pGEX-4T-1 (Amersham, USA) to create GST–OsDERF1 protein in *Escherichia coli* BL21, then the cells were induced by 0.5 mM isopropyl-b-D-thiogalactoside (IPTG) at 16°C for 4 h. Purification of the fusion protein was conducted by affinity chromatography using a Glutathione Sepharose 4B Microspin column according to the manufacturer's instructions (GE Health, USA). Electrophoretic mobility shift assay (EMSA) was performed as described (DIG Gel Shift Kit, Roche, USA). The 3′-DIG-labeled probes were prepared by annealing of the synthesis oligonucleotides listed in Table S4. The binding reactions in 10 mM Tris (pH 7.5), 50 mM KCl, 1 mM DTT, 2.5% glycerol, 0.05% NP-40, 5 mM MgCl_2_, 0.5 mM EDTA, 5 ng/mL poly (dI·dC), 30 ng GST–OsDERF1 recombinant fusion protein, and 1 ng labeled DNA were kept for 15 min at room temperature before loading buffer was added. Gel electrophoresis was performed on a 7% native polyacrylamide gel (29∶1 acryl/bis). After blotting on a positively charged nylon membrane (GE Health, USA), the DNA was linked using a UV light cross-linker instrument for 3 min exposure.

### Quantification of ethylene biosynthesis

Ethylene emission by rice was measured with a gas chromatograph (Shimadzu, Japan) as described [68]. Germinated seedlings were placed in 50-mL glass vials (30 seedlings per vial) containing 3 mL of N_6_B_5_ culture medium with 0.4% phytagel and cultured at 25°C in a growth chamber. After growth for 7 d, all the vials were sealed. Ethylene emission was determined by taking 1 mL volume with an air-tight syringe after 24 h of incubation. Levels of ethylene in the vials were compared to those in control vials that did not contain any seedlings.

## Supporting Information

Figure S1 **Sequence alignment of OsDERF1 protein between japonica and indica varieties.**
(TIF)

Figure S2 **Comparison of Nipponbare and two upland rice varieties in the expression of** ***OsDERF1*** **and drought response.** A. The expression of *OsDERF1* in Nipponbare and upland varieties Zhong Han 3 and Brazil Triphibian. B. Zhong Han 3 and Brazil Triphibian showed better drought tolerant and more ethylene emission than Nipponbare did. The data, average of three independent biological assays plus SD, show ethylene production in terms of the increase over controls (sealed vials without any seedlings). nl/gFW.h indicates the amount of ethylene per gram fresh weight seedling in an hour. C. Knock-down of *OsDERF1* (RI-5) displayed similar drought response with drought tolerant variety Zhong Han 3.
(TIF)

Figure S3 **Subcellular localization of** ***OsDERF1*** **.** Left panel shows the strategy for vector construction, right panel for cellular localization. GFP and the different length of *OsDERF1* with fusion of GFP under the control of the CaMV 35S promoter were expressed transiently in rice callus.
(TIF)

Figure S4 **Developmental observations of** ***OsDERF1*** **transgenic plants under normal growth conditions at seedling and tillering stages.** A. Identification of the *OsDERF1* transgenic rice with RT-PCR (OE lines) and Q-PCR (RI lines), and RI lines did not show any effect on the expression of homolog genes. Rice with reduced expression of *OsDERF1* indicated as RI, overexpressing *OsDERF1* rice as OE, and the different transgenic lines are indicated as the numbers. The expression of *OsDERF1* in the Nipponbare was standardized to 1, referring to the internal control of *OsActin1*. Data are the average of three replicates. Error bars represent standard error (SE). B. Development comparison of RI lines at tillering stage.
(TIF)

Figure S5 **The expression levels of** ***OsP5CS*** **with or without PEG treatment.** The transcripts of *OsP5CS* in the Nipponbare were standardized to 1, referring to the internal control of *OsActin1*. Data are the average of three replicates, and there were 10 plants per replicate. Error bars represent standard error (SE).
(TIF)

Figure S6 **Identification of the** ***OsERF3*** **transgenic rice and overexpressor mutants** ***Oserf3*** **and** ***Osap2-39*** **with Q-PCR.** Overexpressing *OsERF3* in Nipponbare was denoted as OX, and the different transgenic lines are indicated as the numbers. *Oserf3* and *Osap2-39* are activation-tagged T-DNA insertion mutants in Zhonghua 11 (Zh11). The expression level of *OsERF3* and *OsAP2-39* in the Nipponbare or Zh11 was standardized to 1, referring to the internal control of *OsActin1*. Data are the average of three replicates. Error bars represent standard error (SE).
(TIF)

Figure S7 **The phenotype of overexpressor mutants** ***Oserf3*** **and** ***Osap2-39*** **under drought stress.** Drought stress treatment during seedling stage. Plants were subjected to drought stress for 10 d. Control: rice plants were grown under normal conditions. Drought: plants were withheld daily water supply. Each experiment was repeated at least three times, with >30 seedlings each of Zhonghua 11 (Zh11) and overexpressor mutants.
(TIF)

Figure S8 **Detection of the interaction of OsERF3 with GCC box and DRE.** Probe sequences of GCC box and DRE are listed in Table S4. The detection of probes after reaction with GST protein was taken as a negative control. Competition assays were processed using unlabeled probes after reaction with GST–OsERF3 in the presence of labeled probes GCC box or DRE.
(TIF)

Table S1 **ERF genes in response to drought in rice.**
(DOC)

Table S2 **Down-regulated genes of OsDERF1 in Affymetrix GeneChip.**
(XLS)

Table S3 **Up-regulated genes of OsDERF1 in Affymetrix GeneChip.**
(XLS)

Table S4 **The primers and oligonucleotides used in this paper.**
(XLS)

Table S5 **Analyses of cis-acting elements in the promoters of rice ACO and ACS genes.**
(DOC)
