# Supplementary figures and images for: Transcriptional Activation of OsDERF1 in OsERF3 and OsAP2-39 Negatively Modulates Ethylene Synthesis and Drought Tolerance in Rice
Source: PLoS One. 2011 Sep 26;6(9):e25216. doi: 10.1371/journal.pone.0025216 (PMC3180291; doi:10.1371/journal.pone.0025216)

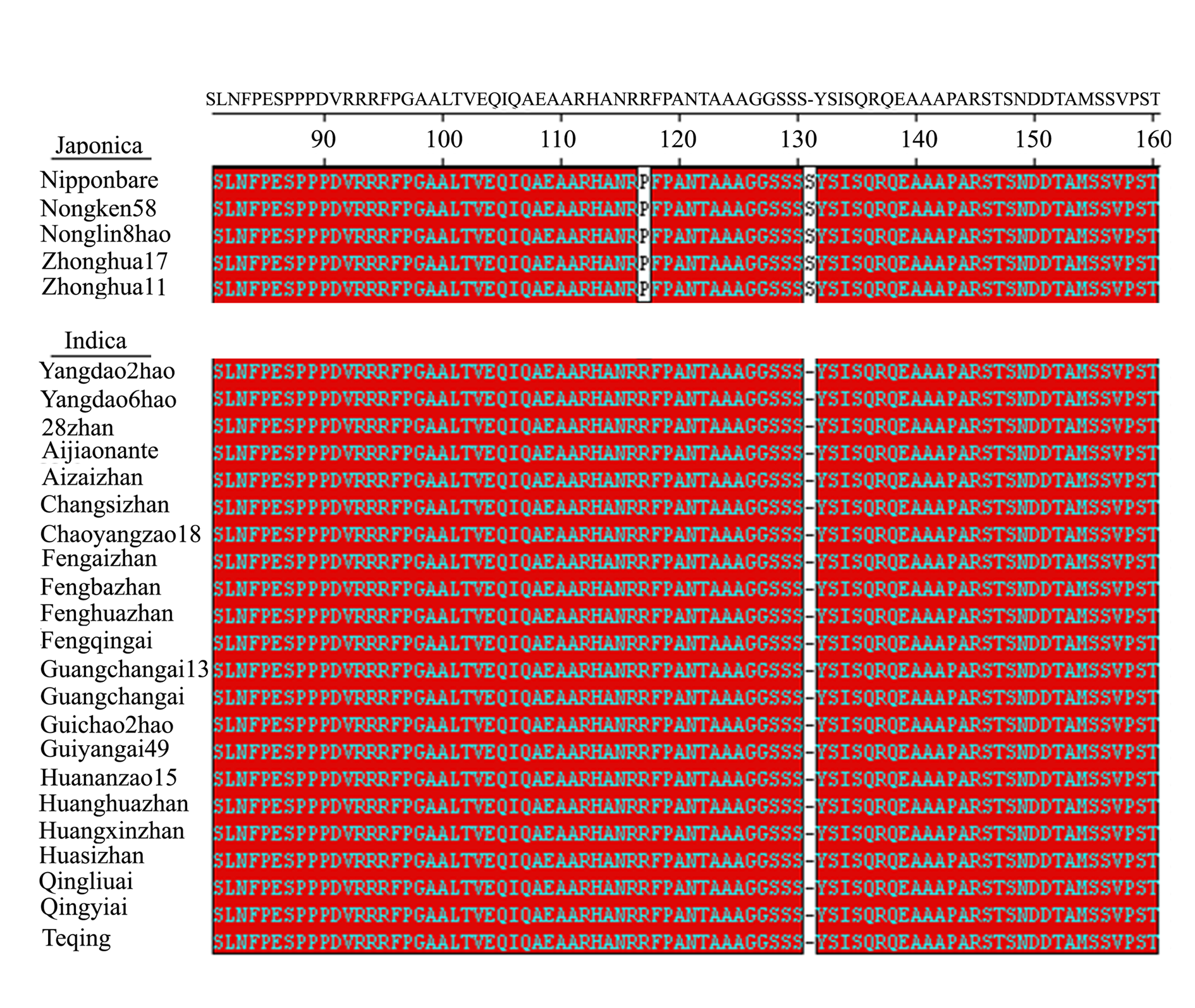

Supplement: Figure S1 — Sequence alignment of OsDERF1 protein between japonica and indica varieties. (TIF) [file pone.0025216.s001.tif]

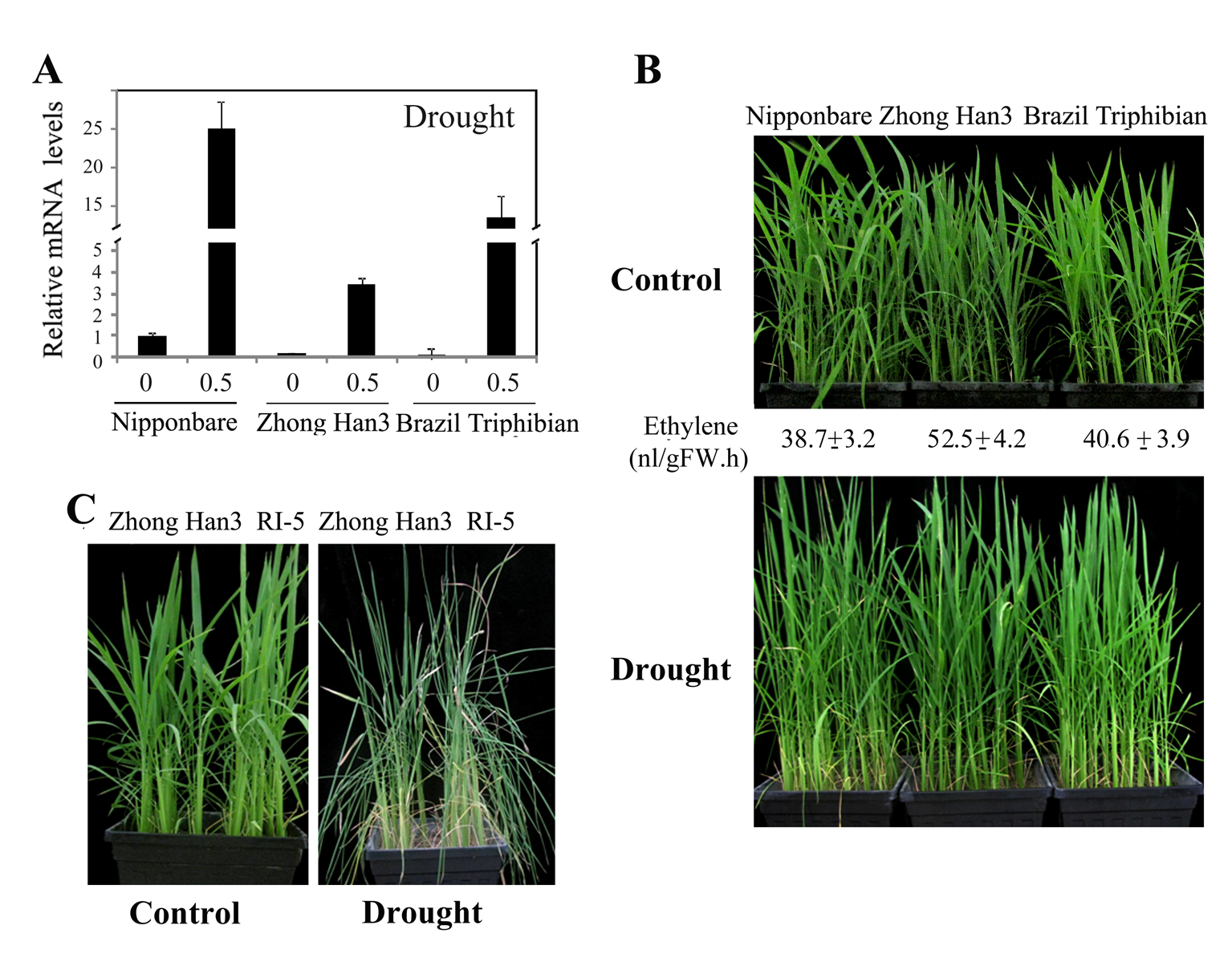

Supplement: Figure S2 — Comparison of Nipponbare and two upland rice varieties in the expression of OsDERF1 and drought response. A. The expression of OsDERF1 in Nipponbare and upland varieties Zhong Han 3 and Brazil Triphibian. B. Zhong Han 3 and Brazil Triphibian showed better drought tolerant and more ethylene emission than Nipponbare did. The data, average of three independent biological assays plus SD, show ethylene production in terms of the increase over controls (sealed vials without any seedlings). nl/gFW.h indicates the amount of ethylene per gram fresh weight seedling in an hour. C. Knock-down of OsDERF1 (RI-5) displayed similar drought response with drought tolerant variety Zhong Han 3. (TIF) [file pone.0025216.s002.tif]

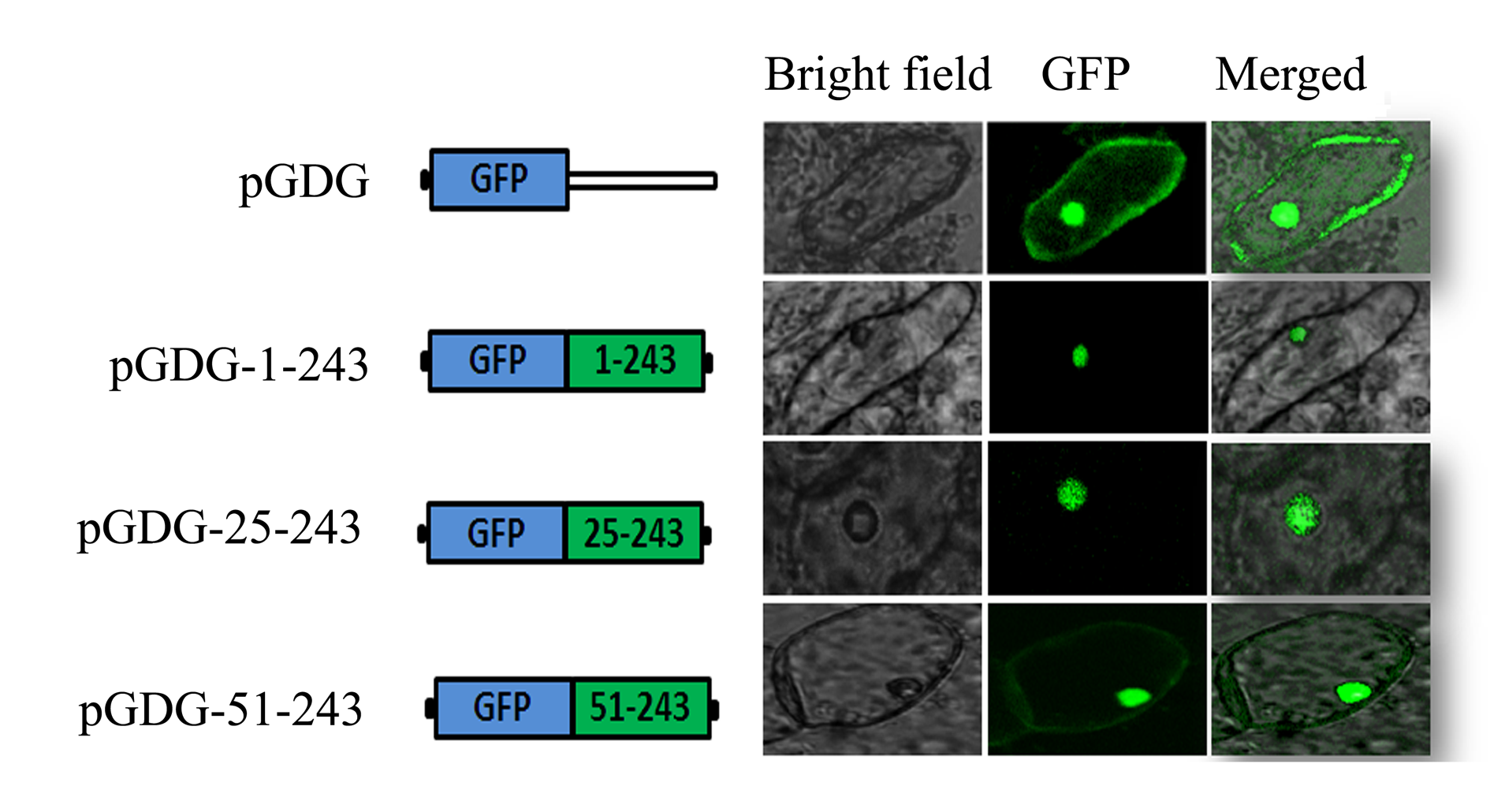

Supplement: Figure S3 — Subcellular localization of OsDERF1 . Left panel shows the strategy for vector construction, right panel for cellular localization. GFP and the different length of OsDERF1 with fusion of GFP under the control of the CaMV 35S promoter were expressed transiently in rice callus. (TIF) [file pone.0025216.s003.tif]

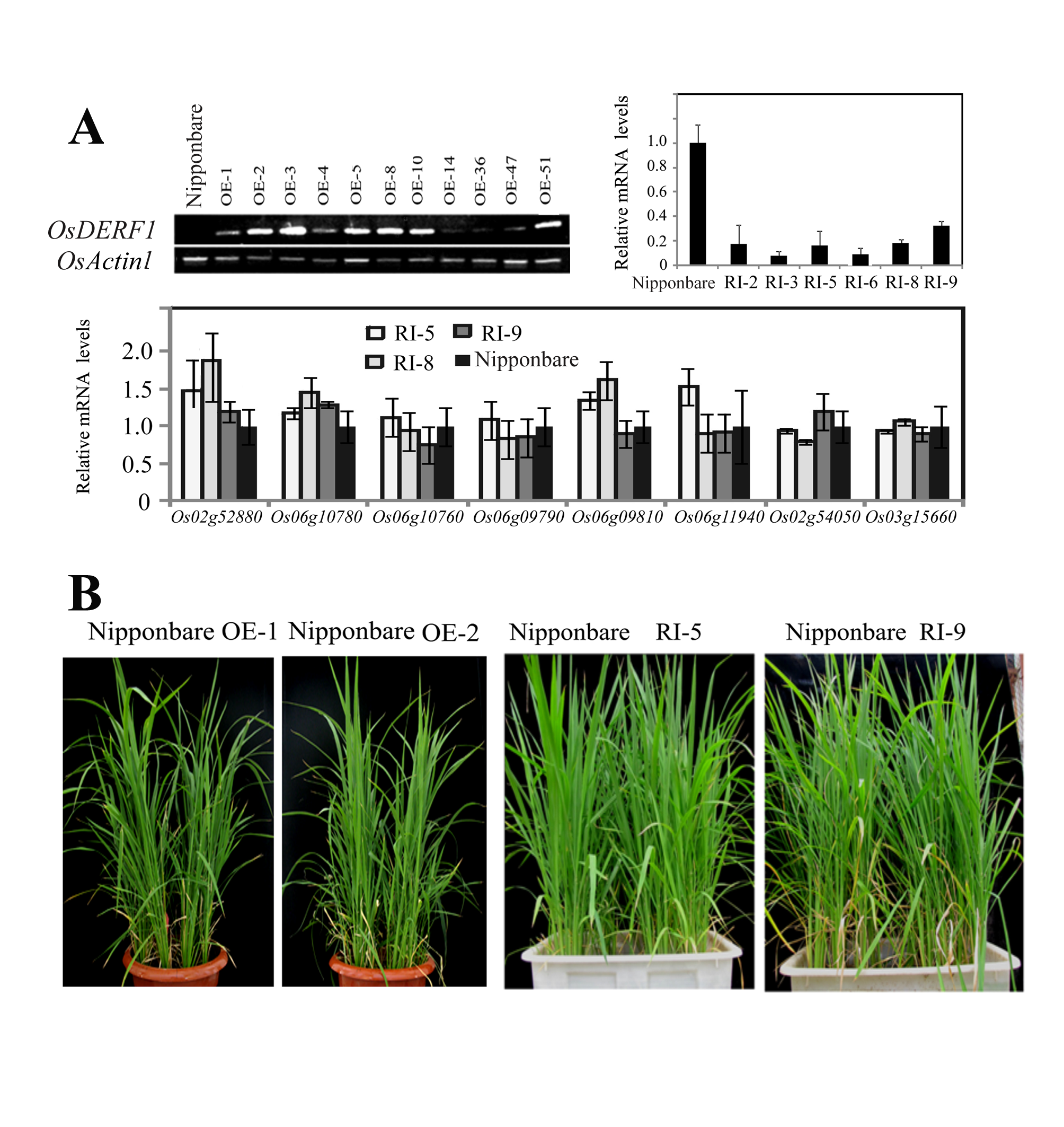

Supplement: Figure S4 — Developmental observations of OsDERF1 transgenic plants under normal growth conditions at seedling and tillering stages. A. Identification of the OsDERF1 transgenic rice with RT-PCR (OE lines) and Q-PCR (RI lines), and RI lines did not show any effect on the expression of homolog genes. Rice with reduced expression of OsDERF1 indicated as RI, overexpressing OsDERF1 rice as OE, and the different transgenic lines are indicated as the numbers. The expression of OsDERF1 in the Nipponbare was standardized to 1, referring to the internal control of OsActin1. Data are the average of three replicates. Error bars represent standard error (SE). B. Development comparison of RI lines at tillering stage. (TIF) [file pone.0025216.s004.tif]

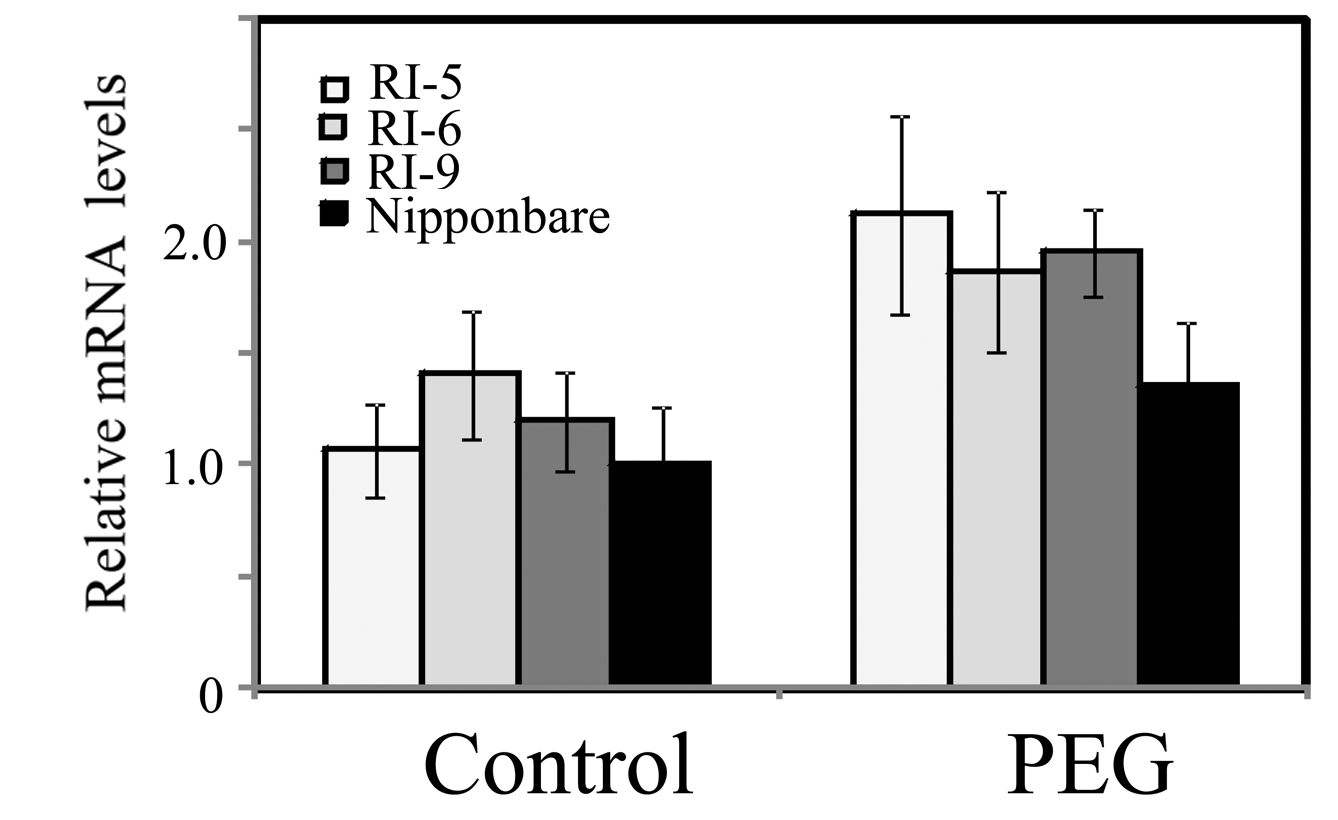

Supplement: Figure S5 — The expression levels of OsP5CS with or without PEG treatment. The transcripts of OsP5CS in the Nipponbare were standardized to 1, referring to the internal control of OsActin1. Data are the average of three replicates, and there were 10 plants per replicate. Error bars represent standard error (SE). (TIF) [file pone.0025216.s005.tif]

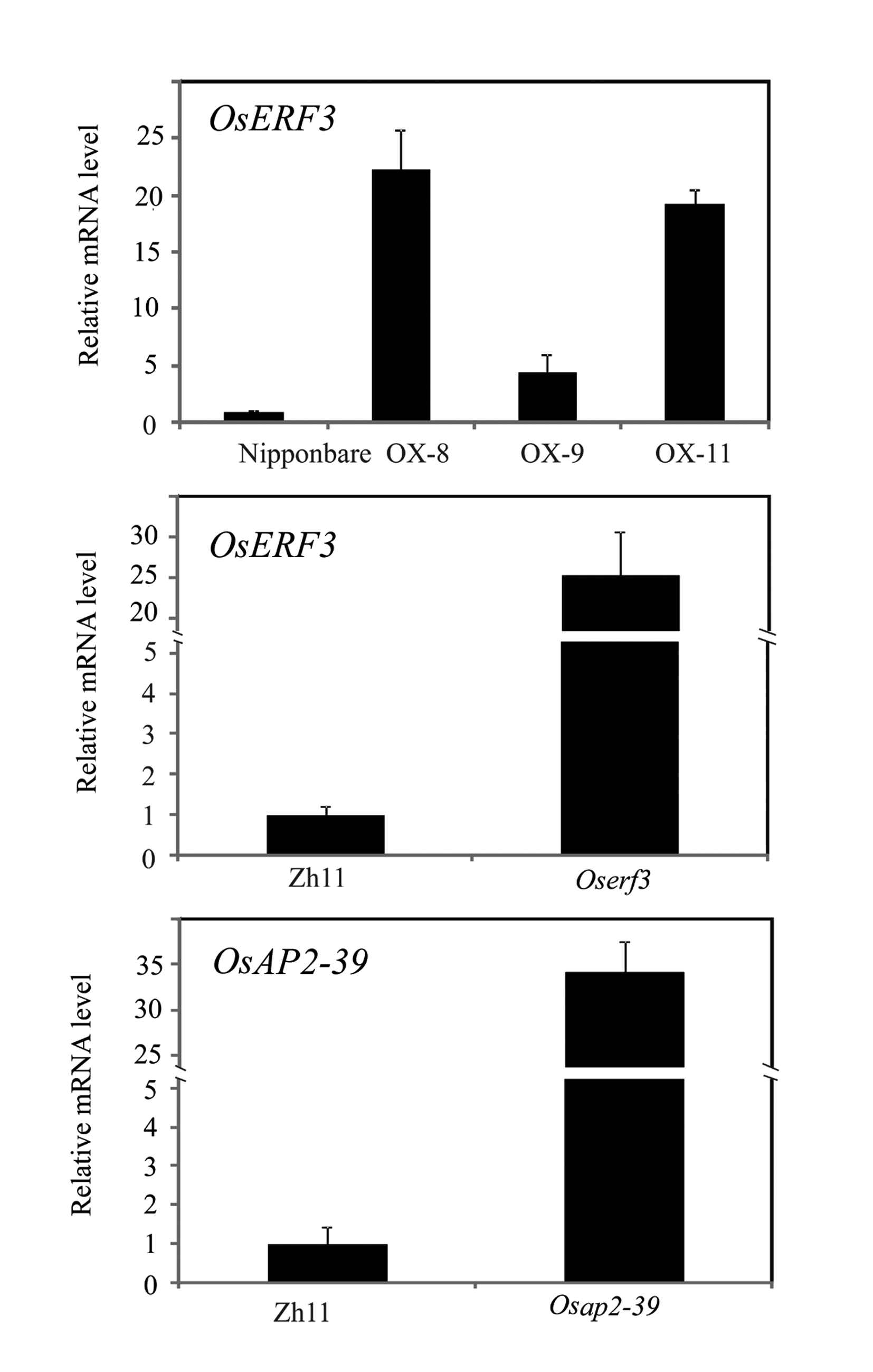

Supplement: Figure S6 — Identification of the OsERF3 transgenic rice and overexpressor mutants Oserf3 and Osap2-39 with Q-PCR. Overexpressing OsERF3 in Nipponbare was denoted as OX, and the different transgenic lines are indicated as the numbers. Oserf3 and Osap2-39 are activation-tagged T-DNA insertion mutants in Zhonghua 11 (Zh11). The expression level of OsERF3 and OsAP2-39 in the Nipponbare or Zh11 was standardized to 1, referring to the internal control of OsActin1. Data are the average of three replicates. Error bars represent standard error (SE). (TIF) [file pone.0025216.s006.tif]

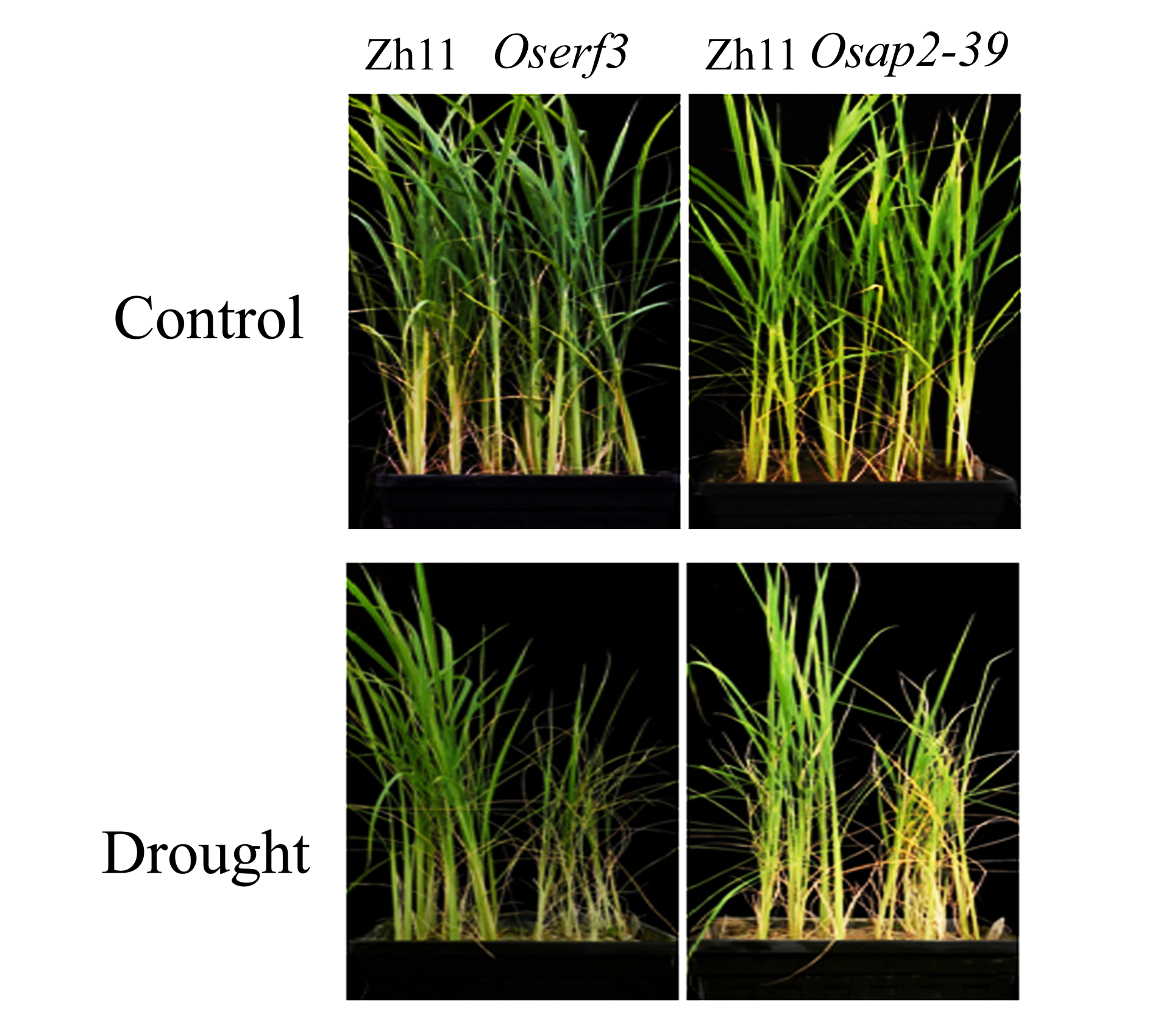

Supplement: Figure S7 — The phenotype of overexpressor mutants Oserf3 and Osap2-39 under drought stress. Drought stress treatment during seedling stage. Plants were subjected to drought stress for 10 d. Control: rice plants were grown under normal conditions. Drought: plants were withheld daily water supply. Each experiment was repeated at least three times, with >30 seedlings each of Zhonghua 11 (Zh11) and overexpressor mutants. (TIF) [file pone.0025216.s007.tif]

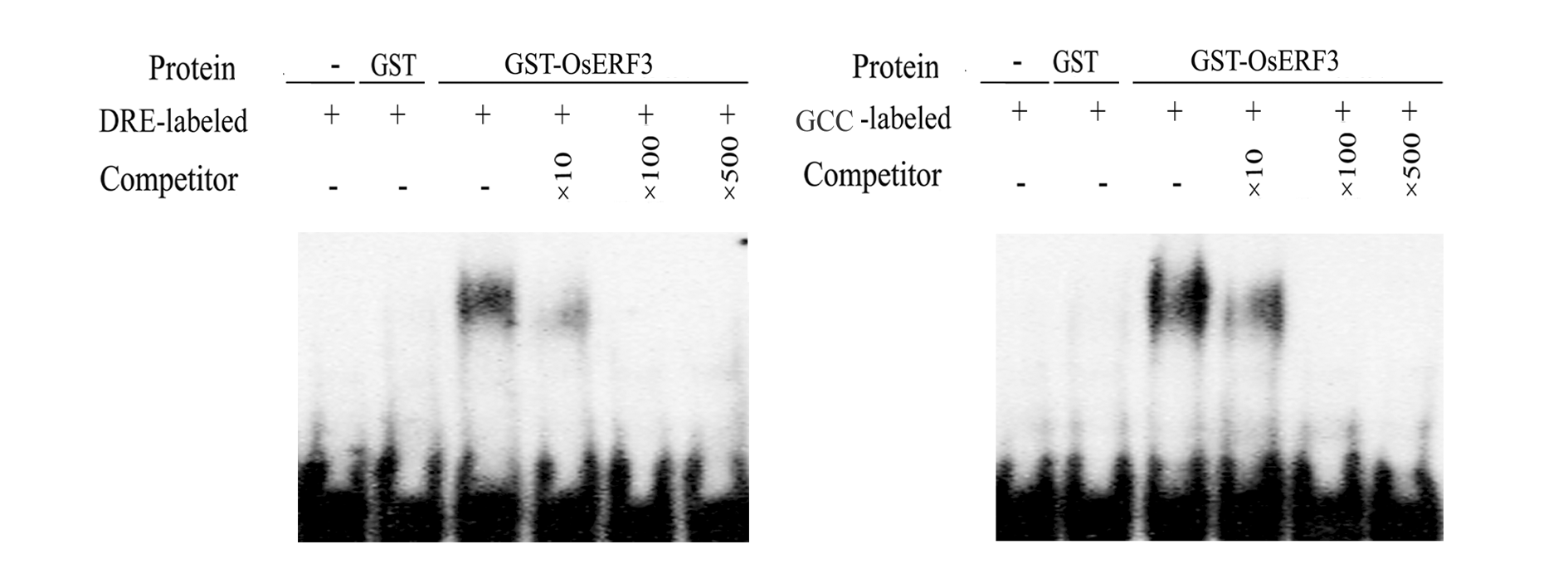

Supplement: Figure S8 — Detection of the interaction of OsERF3 with GCC box and DRE. Probe sequences of GCC box and DRE are listed in Table S4. The detection of probes after reaction with GST protein was taken as a negative control. Competition assays were processed using unlabeled probes after reaction with GST–OsERF3 in the presence of labeled probes GCC box or DRE. (TIF) [file pone.0025216.s008.tif]
