# Supplementary material for: Transcriptional Activation of OsDERF1 in OsERF3 and OsAP2-39 Negatively Modulates Ethylene Synthesis and Drought Tolerance in Rice
Source: PLoS One. 2011 Sep 26;6(9):e25216. doi: 10.1371/journal.pone.0025216 (PMC3180291; doi:10.1371/journal.pone.0025216)
Supplement: Table S1 — ERF genes in response to drought in rice. (DOC) [file pone.0025216.s009.doc]

**S Table 1: Up-regulated ERF genes in response to drought in rice**

| AGI ID | Expression tissue | Description | cDNA accession No |
| --- | --- | --- | --- |
| Os08g35240  (OsDERF1) | Leaf | OsERF#012 | -- |
| Os07g22770  (OsDERF2) | Leaf | OsERF#086 | AK062612 |
| Os08g36920  (OsDERF3) | Leaf | OsERF#104 | AK062882 |
| Os08g43200  (OsDERF4) | Leaf | OsERF#028 | -- |
| Os02g52670  (OsDERF5) | Leaf | OsERF#103 | AK107146 |
| Os04g32620  (OsDERF6) | Leaf | OsERF#101 | AK287453 |
| Os08g42550  (OsDERF7) | Leaf | OsERF#106 | AK064252 |
| Os12g07030  (OsDERF8) | Root | OsERF#124 | AK107125 |
| Os02g54050  (OsDERF9) | Root | OsERF#011 | -- |
| Os05g49010  (OsDERF10) | Root | OsERF#084 | -- |

| Os10g22600  (OsDERF11) | Root | OsERF#051 | -- |
| --- | --- | --- | --- |
| Os10g41330  (OsDERF12) | Root | OsERF#096 | -- |

The data were from e[xpression data for stress treatment in rice seedlings](../xpression%20data%20for%20stress%20treatment%20in%20rice%20seedlings) ([http://www.ricearray.org](http://www.ricearray.org/), GSE26280).

The description of genes was from Nakano et al. 2006, Plant Physiol. 140: 411-432.
