# Supplementary material for: Transcriptional Activation of OsDERF1 in OsERF3 and OsAP2-39 Negatively Modulates Ethylene Synthesis and Drought Tolerance in Rice
Source: PLoS One. 2011 Sep 26;6(9):e25216. doi: 10.1371/journal.pone.0025216 (PMC3180291; doi:10.1371/journal.pone.0025216)
Supplement: Table S5 — Analyses of cis-acting elements in the promoters of rice ACO and ACS genes. (DOC) [file pone.0025216.s013.doc]

S Table 4: Cis-elements in the promoters of rice ACO and ACS genes

| Gene | | Locus | | cDNA | Cis-element in promotor |
| --- | --- | --- | --- | --- | --- |
| *OsACS1* | Os03g51740 | | AK071011 | | DRE |
| *OsACS2* | Os04g48850 | | AK064250 | | GCC box |
| *OsACS3* | Os05g10780 | | P0617H07.9 | |  |
| *OsACS4* | Os05g25490 | | OSJNBb0006B22.3 | | GCC box |
| *OsACS5* | Os01g09700 | | D46839 | | DRE |
| *OsACS6* | Os06g03990 | | AK065212 | | DRE\GCC box |
| *OsACO1* | Os09g27820 | | AK058296 | |  |
| *OsACO2* | Os02g53180 | | AK071557 | | DRE\GCC box |
| *OsACO3* | Os09g27750 | | AK065039 | | DRE\GCC box |
| *OsACO4* | Os11g08380 | | AK105491 | | GCC box |
| *OsACO5* | Os05g05680 | | AK061064 | |  |
| *OsACO6* | Os05g05670 | |  | | DRE |
| *OsACO7* | Os01g39860 | | AK102472 | | DRE |
